# Characteristics of COVID-19 (Delta Variant)/HIV Co-infection: A Cross-sectional Study in Henan Province, China

**DOI:** 10.1007/s44231-022-00018-z

**Published:** 2022-11-15

**Authors:** Rui Yang, Jiuling Cheng, Xiangjin Song, Yuanwei Pan, Huaqi Wang, Jing Li, Xudong He, Jianjun Gou, Guojun Zhang

**Affiliations:** 1 Department of Respiratory Medicine, The First Affiliated Hospital of Zhengzhou University, No.1, Jianshe East Road, Zhengzhou, 450052 Henan People’s Republic of China; 2 Department of Radiology, The First Affiliated Hospital of Zhengzhou University, Zhengzhou, 450052 Henan People’s Republic of China; 3 Department of General Surgery, The First Affiliated Hospital of Zhengzhou University, No.1, Jianshe East Road, Zhengzhou, 450052 Henan People’s Republic of China

**Keywords:** COVID-19, SARS-CoV-2, Delta variant, HIV

## Abstract

**Background:**

Since the end of July 2021, SARS-CoV-2 (Delta variant) invaded Henan Province, China, causing a rapid COVID-19 spread in the province. Among them, the clinical features of COVID-19 (Delta Variant)/HIV co-infection have attracted our attention.

**Methods:**

We included 12 COVID-19 patients living with HIV (human immunodeficiency virus) from July 30, 2021 to September 17, 2021 in Henan Province, China. Demographic, clinical, laboratory, and computed tomography (CT) imaging data were dynamically collected from first nucleic acid positive to hospital discharge. Laboratory findings included SARS-CoV-2 viral load, HIV viral load, IgM, IgG, cytokines, lymphocyte subpopulation, ferritin, etc. Statistical analyses were performed using IBM SPSS version 26·0 and GraphPad Prism version 9·0.

**Results:**

It was founded that the low Ct value persisted for about 21 days, and the viral shedding time (turn negative time) of the patients was 32·36 ± 2·643 days. Furthermore, chest CT imaging revealed that lesions were obviously and rapidly absorbed. It was surprising that IgM levels were statistically higher in patients taking azvudine or convalescent plasma than in patients not taking these drugs (*P* < 0·001, *P* = 0·0002, respectively). IgG levels were significantly higher in patients treated with the combined medication of BRII/196 and BRII/198 than in those not treated with these drugs (*P* = 0·0029). IgM was significantly higher in those with low HIV viral load than those with high HIV viral load (*P* < 0·001). In addition, as treatment progressed and patients' condition improved, IL-17a showed a decreasing trend.

**Conclusions:**

Based on this study, we found that HIV infection might not exacerbate COVID-19 severity.

**Supplementary Information:**

The online version contains supplementary material available at 10.1007/s44231-022-00018-z.

## Background

Coronavirus disease 2019 (COVID-19) is an acute respiratory infectious disease caused by severe acute respiratory syndrome coronavirus 2 (SARS-CoV-2) [1]. The clinical manifestations of COVID-19 are highly variable in severity, ranging from asymptomatic carriage to severe respiratory disease and death [2, 3]. The virus has spread rapidly worldwide [4, 5] with over 237 million confirmed cases and more than 4 million deaths as of October 11, 2021 [6], In terms of number of infections and area of transmission, it has far surpassed Middle East Respiratory Syndrome (MERS) and Severe Acute Respiratory Syndrome (SARS) [7, 8]. The COVID-19 outbreak poses a serious threat to global public health and is a major challenge to the response of HIV-infected communities to the COVID -19 crisis.

A much debated question is the outcome of the patients infected with SARS-CoV-2 and HIV. Several studies have shown that AIDS patients infected with SARS-CoV-2 have a higher risk of hospitalization and a worse clinical prognosis [9–13]. However, there are conflicting reports on this. That is, compared with the general population, people living with HIV (PLWH) are not at higher risk than the general population, provided that the CD4^+^T cell count is greater than 200 cells/ml and viral replication is controlled by antiretroviral treatment [14–17]. The prognosis of COVID-19/HIV co-infection is not uniform, and the association between HIV and SARS-CoV-2(Delta variant) has not been described. There is a need for more clinical data and analysis to form the basis for further mechanistic studies.

The spike protein (S) [18] of SARS-COV-2 binds to ACE2 (angiotensin-converting enzyme 2) on the surface of airway epithelial cells, mediated by cathepsin L and the transmembrane protease serine 2 (TMPRSS2) [4, 5, 19], making the affinity of SARS-CoV-2 for ACE2 10- to 20-fold higher than that of SARS [20]. However, since the SARS-COV-2 Delta variant (B·1·617·2) was first identified in India in December 2020 [21–24], it has rapidly become the dominant variant in many countries [23, 25, 26]. Studies have shown that the risk of hospitalization is more than twofold higher for delta variants than for alpha variants [25, 27, 28]. The viral load of Delta variant was significantly higher than that of the previous variants [29], and it has a high transmission rate, increased immune escape, and increased risk of disease progression [30]. There are currently no highly relevant reports on the outcome of SARS-CoV-2 (Delta variant)/HIV co-infection and the relationship between Delta Variant and HIV remains unclear [31].

In July 2021, a new round of COVID-19 epidemics broke out in Henan Province, China. After sequencing the virus in the laboratory of the Provincial Center for Disease Control and Prevention and comparing and analyzing it with the virus strains of recent imported cases, this epidemic was mainly caused by SARS-CoV-2 (delta variant). Among the confirmed patients, those with COVID-19/HIV co-infection have almost no clinical symptoms of viral pneumonia and the uptake of lesions in the chest CT imaging is rapid and complete, which attracted our attention. In addition, the clinical features, molecular immune characteristics and prognosis of patients coinfected with Delta variant and HIV are still unclear. To describe the above points, this article will summarize in detail the clinical characteristics of patients co-infected with delta variant and HIV, SARS-CoV-2 (Delta) viral loads, antibodies, lymphocyte subsets, cytokines and other test indicators dynamically, and then analyze their relationships with HIV viral loads and analyze the immune-related effects of drugs in eliminating SARS-CoV-2, providing valuable clinical evidence and new ideas for the studies of COVID-19(Delta)/HIV co-infected patients.

## Methods

### Patients and Data Collection

All confirmed patients of this outbreak in Henan, China, were transferred to Airport Zone Hospital of Zhengzhou First People's Hospital or special treatment of COVID -19. We enrolled all confirmed cases with COVID-19/HIV co-infection admitted to the designated hospital (Airport Zone Hospital of Zhengzhou First People's Hospital) in Henan Province, China from July 30, 2021 to September 17, 2021 in this prospective study. Thirteen cases were eligible, including one case of absence, and finally a total of 12 confirmed cases were included. Relevant indicators were monitored at intervals of 3–7 days. We then analyzed the dynamic changes in the indices. All clinical data (including basic information, clinical manifestations, laboratory findings, treatments, and outcomes during hospitalization) were obtained from patients' electronic medical records. The mean duration of clinical observation was 36·42 ± 2·148 days, and the longest was 46 days. Patients were diagnosed and treated according to the "Diagnostic and treatment protocol for Novel Coronavirus Pneumonia (the eighth edition)" issued by the National Health Commission of the People's Republic of China [32].

### SARS-CoV-2 RNA Measurement

SARS-CoV-2 RNA load in nasal swabs was measured by RT-qPCR using primers and probes targeting the SARS-CoV-2 ORF1ab and N gene and detected using the 2019-nCoV Nucleic Acid Test Kit (bioperfectus, technologies). Ct = 37 is the cutoff for a positive result and Ct = 40 is a negative sample; Ct = 40 was the limit of detection. The diagnostic criteria were in accordance with the recommendations of the National Institute for Viral Disease Control and Prevention in China.

### Serologic Testing

IgM and IgG antibodies against SARS-CoV-2 and ferritin were determined by semi-quantitative magnetic particle chemiluminescence immunoassays (M-CLIAs) (Autobio, Zhengzhou, China). Antibody levels were expressed as measured chemiluminescence values divided by the cutoff value (S/Co).

Cytokines (IL-6, IL-12p70, IL-4, IL-17a, IL-10, IL-2, IFN-r, TNF-alpha) were detected by Cytokine Combination Assay Kit (Immunofluorescence) (cell-genebio, Jiangxi, China).

Lymphocyte subsets were measured by Bricyte E6 flow cytometer (Mindray, China).

### Statistical Analysis

Statistical analyses were performed using IBM SPSS version 26·0 and GraphPad Prism version 9·0. Data were presented as numbers and frequencies for categorical data. After normality test using Shapiro–Wilk test, continuous variables of normal distribution were represented by mean ± SD, and data that did not conform to normal distribution were represented as median (interquartile range [IQR]: Q1, Q3). Statistical comparisons between groups were performed using the Mann–Whitney *U* test for continuous variables. Correlations were analyzed using the Spearson test. A *P* value of less than 0·05 was considered statistically significant.

## Results

### Demographic and Clinical Features of the Study Population

Of the 167 patients with COVID-19 who had been hospitalized at designated sites as of July 30, 2021 in this outbreak of COVID-19 [33], we found 13 (7·8%) cases who were COVID-19 patients living with HIV, of which there was one abscission case, and finally a total of 12 (7·2%) confirmed cases were included data. There were 8 men and 4 women in the study participants. The median age of COVID/HIV co-infection patients is 48 years old (range 33·5–51); The patients were classified into four clinical groups based on disease severity at admission, namely mild (7/12, 58%), moderate (5/12, 42%), severe (0/12, 0%), or critical (0/12, 0%); and then as the course of the disease progresses, the current case classification is that the ratio of mild, moderate, severe and critical is 2:10:0:0; All patients were discharged after treatment. There is no significant difference in length of hospital stay or prognosis between HIV patients and non-HIV patients (Fig. S1). Except for 1 patient with nontuberculous mycobacterial infection, persistent fever, multiple lymphadenopathy throughout the body, and 1 case of hyposmia, the remaining cases did not have fever, cough, sputum, chest tightness, hypoosmia and other viral pneumonia. The performance has not changed much compared with before the infection of the new coronavirus. Except for one case with non-tuberculous mycobacterium infection, who had persistent fever, generalized lymphadenopathy, and one patient with hyposmia, the remaining cases showed no specific clinical manifestation of viral pneumonia such as fever, cough, expectoration, chest tightness, which didn’t change much compared with those before SARS-CoV-2 infection. The average baseline blood cell counts at admission were within the normal range (the lymphocytes in 4 confirmed cases, hemoglobin in 4 patients and platelet in 2 ones were lower than normal) (Table 1). The comorbidities of confirmed cases are numerous and severe, and some patients have as many as five comorbidities. None of the study subjects were vaccinated, and the effect of antiviral treatment was not significant.

**Table 1:** Baseline characteristics in all patients with COVID-19/HIV- coinfection

Variables	Total (*n* = 12)
Gender (*n*, %)	
Male	8 (67%)
Female	4 (33%)
Age (y), median (Q1, Q3)	48 (33·5, 51)
Complete Blood Count, median (Q1, Q3)	
WBC (× 10^9^/L)	3·56 (2·96, 5·26)
Neutrophils (× 10^9^/L)	2·48 (1·73, 2·81)
Lymphocytes (× 10^9^/L)	1·19 (0·76, 1·65)
Monocytes (× 10^9^/L)	0·31 (0·21, 0·40)
Eosinophils (× 10^9^/L)	0·09 (0·02, 0·17)
Basophils (× 10^9^/L)	0·02 (0·01, 0·03)
Hemoglobin (g/L)	121·5 (94·75, 128·75)
Platelet (× 10^9^/L)	157 (134, 207)
d-Dimmer	1·075 (0·4075, 1·590)
Coexisting disorders (*n*, %)	
AIDS	12 (100%)
Hepatitis B	1 (8%)
Hepatitis C	2 (17%)
Syphilis	1 (8%)
Hypertension	1 (8%)
Diabetes	2 (17%)
Renal insufficiency	2 (17%)
NTM	1 (8%)
Vaccination (*n*, %)	
Yes	0 (0%)
No	12 (100%)
Admission clinical classification (*n*, %)	
Mild	7 (58%)
Moderate	5 (42%)
Severe	0 (0%)
Critical	0 (0%)
Treatment—no, %	
Oxygen inhalation	2 (17%)
Chinese medicine	12 (100%)
Antiretroviral treatment (ART)	12 (100%)
Low molecular weight heparins calcium	10 (83%)
Intravenous immunoglobulin	7 (58%)
Azvudine	6 (50%)
Thymalfasin	11 (92%)
Convalescent plasma	6 (50%)
Combined medication of BRII/196 and BRII/198	8 (67%)
IFN-α	1 (8%)
IL-2	6 (50%)
Clinical outcome	
Hospital discharge	11 (92%)
Death	0 (0%)

*AIDS* Acquired Immune Deficiency Syndrome, *NTM* nontuberculous mycobacteria; *IFN* interferon, *IL* interleukin

During hospitalization, 2 patients (17%) received nasal catheter oxygen inhalation, and patients received Traditional Chinese medicine (12, 100%), antiretroviral therapy (12, 100%), low molecular weight heparin (10, 83%), Intravenous immunoglobulin (7, 58%), Azvudine (6, 50%), Thymalfasin (11, 92%), convalescent plasma (6, 50%), combined medication of BRII/196 and BRII/198 (8, 67%), IFN-α (1, 8%), IL-2 (6, 50%). None of the patients received systemic corticosteroids (Table 1).

### Laboratory Findings/Laboratory and Radiographic Findings

We screened out 12 COVID-19 patients living with HIV from the electronic medical record. Since first nucleic acid positive, we observed their clinical symptoms, chest CT features, laboratory indexes, including SARS-CoV-2 viral load, IgM, IgG, Cytokines, lymphocyte subpopulation, ferritin, etc. We monitored he above indicators and record them at intervals of 3–7 days. The average observation time of patients was 36·42 ± 2·148 days, and the longest was 46 days.

### The Evolution of Lesions Among the Chest CT Examinations

**Fig. 1:**
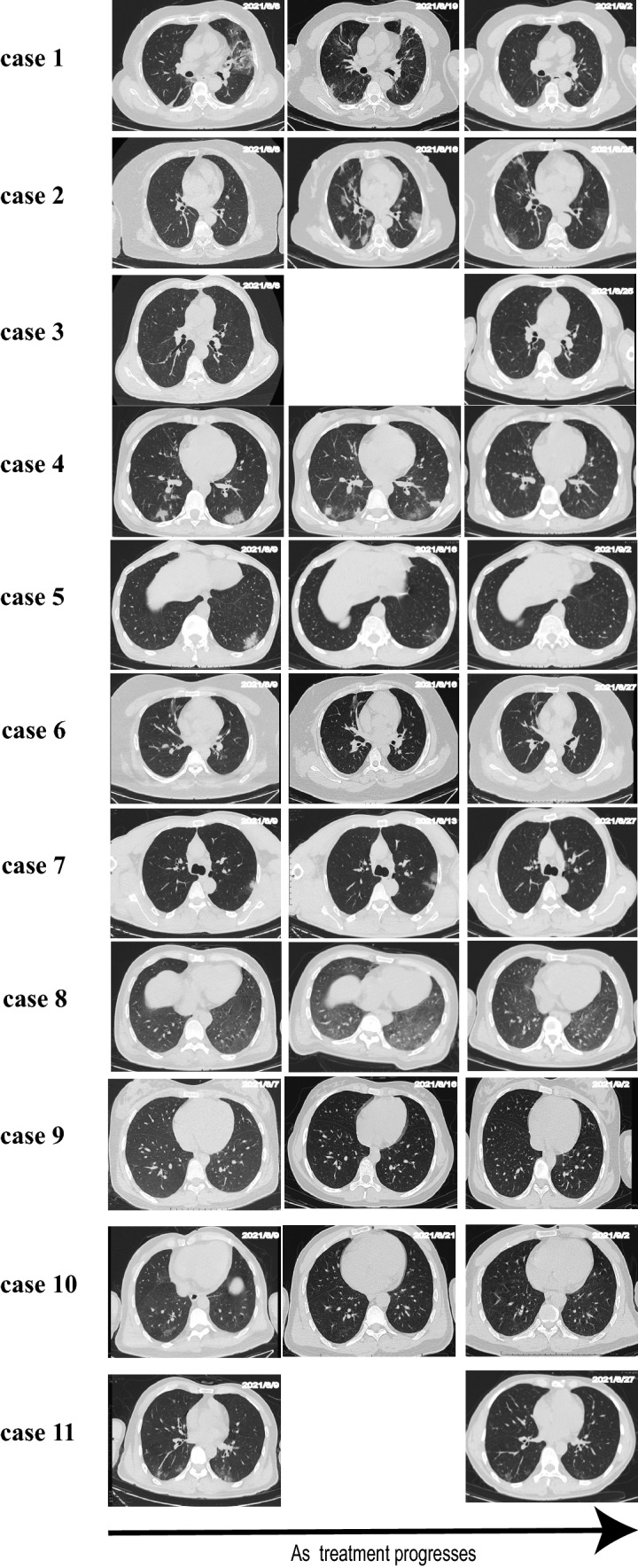
Evolution of the lesions on chest CT of patients with COVID-19/HIV co-infection. As treatment progresses, the lesions f CT images gradually disappear. CT of one case is absent

Chest CT imaging revealed that lesions absorption was obvious and quick, most of which have no sequelae. Figure 1 reveals the comparison of CT imaging of 12 cases scanned at hospital admission and discharge.

### Dynamic Change of SARS-CoV-2 Delta Variant Viral Load

**Fig. 2:**
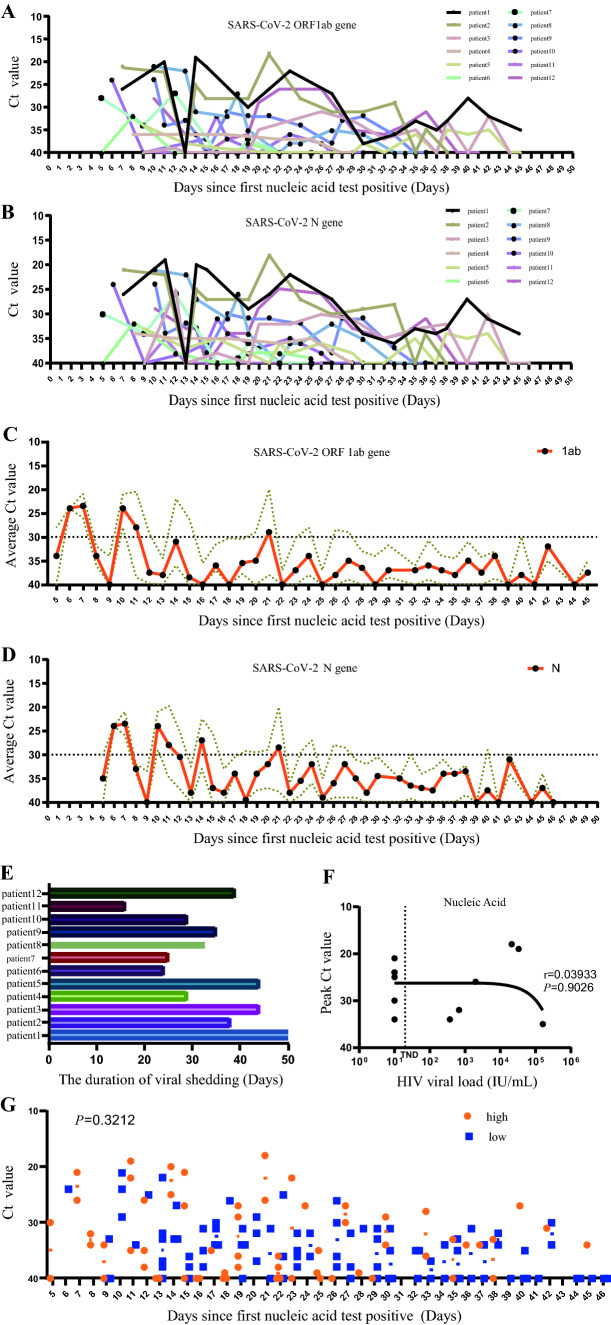
Changes in the Ct value targeting at SARS-CoV-2 ORF1ab (**A**) and N (**B**) gene of RT-qPCR in 12 patients with COVID-19/HIV co-infection. Ct = 37 is the cut-off for a positive result and Ct = 40 is a negative sample; Ct = 40 was the limit of detection. **C** represents Mean Ct value targeting at SARS-CoV-2 1ab gene from all patients at the different time interval between the first positive nucleic acid test, **D** represents Ct value targeting at SARS-CoV-2 N gene from all patients at the different time interval between the first positive nucleic acid test, Brown dotted lines represent error bar. **E** shows that the viral shedding time of COVID-19/HIV co-infection. **F** shows the correlation between Peak Ct value of SARS-CoV-2 N gene and HIV viral load, TND in X axis as an acronym for target not detected. **G** analyzes the difference of the SARS-CoV-2 Ct value between the two groups (< 200 and ≥ 200 copies/ml) of patients at different times since the first nucleic acid test positive

All patients received nasopharyngeal swab sampling at regular intervals and were tested by real-time reverse transcriptase-polymerase chain reaction assays (Fig. 2A, B). Since first SARS-Cov2 nucleic acid test Positive, we tracked and analyzed average cycle threshold (Ct) value in all patients every day. We found low Ct value for about 21 days, indicating the median viral load was greater for a long period (Fig. 2C, D). By the end of the observation period, there was still 1 patient whose nucleic acid had not turned negative, and the virus shedding time (turning negative time) of the remaining 11 patients was 32·36 ± 2·643 days (Fig. 2E). The correlation of viral load between SARS-CoV-2 and HIV is *r* = 0·03,933 (*P* = 0·9026), indicating that SARS-CoV-2 viral load has nothing to do with HIV viral load (Fig. 2F); HIV viral loads of patients were 10, 10, 10, 10, 10, 10, 374, 678, 2000, 21,200, 33,300 and 162,000, respectively. We selected the median HIV viral load of the patients as the cutoff value, The HIV viral load was divided into two groups, and the difference of the SARS-CoV-2 Ct value between the two groups of patients at different times since the first nucleic acid test positive, we found that there was no statistical significance. (*P* = 0·3212) (Fig. 2G).

### The Relationship Between Treatment, HIV Viral Load and Antibody Levels

**Fig. 3:**
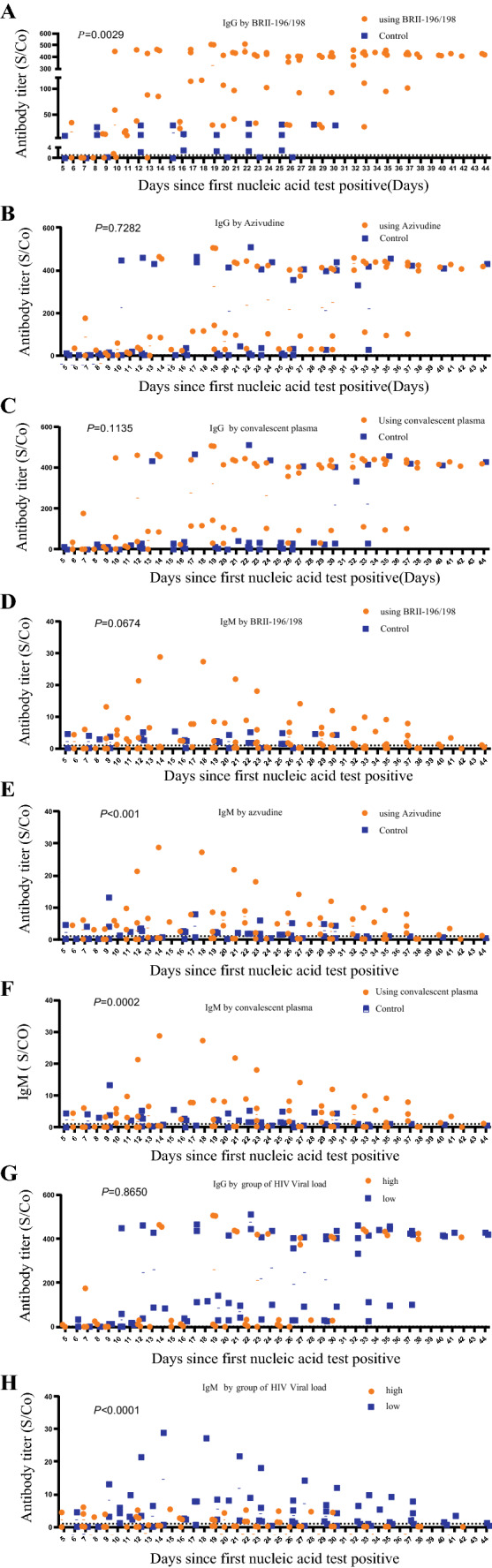
**A**–**C** represents the changes in IgG levels in combined medication of BRII/196 and BRII/198, Azvudine, Convalescent plasma, respectively. **D**–**F** represents the changes in IgM levels in combined medication of BRII/196 and BRII/198, Azvudine, Convalescent plasma, respectively. **G** shows the difference of IgG changes between two groups of HIV viral load, H represents difference of IgM changes between two groups of HIV viral load. X axis represents the interval time from the first positive nucleic acid test

IgM and IgG of these patients were detected regularly to observe their dynamic changes. We found that three cases were negative for IgM and IgG before using the combined medication of BRII/196 and BRII/198, however, after using that, although the IgG increased sharply, IgM was not produced until hospital discharge. The effects of different treatment groups on IgM and IgG repeated measurements were compared. We observed that the differences between azvudine, convalescent plasma and IgG of all cases are not statistically significant (*P* = 0·7282, *P* = 0·1135, respectively) (Fig. 3B, C). Intriguingly, IgG levels were significantly higher in patients treated with the combined medication of BRII/196 and BRII/198 than in those not treated with these. (*P* = 0·0029) (Fig. 3A); However, there was no significant difference between IgM levels by using the combined medication of BRII/196 and BRII/198(*P* = 0·0674) (Fig. 3D). It was surprising that IgM of cases using Azvudine or convalescent plasma were statistically higher than those in patients who did not have a dispensing of these medicines (*P* < 0·001, *P* = 0·0002, Respectively) (Fig. 3E, F). The HIV viral load was analyzed into two groups (< 200 and ≥ 200 copies/ml),namely low group and high group, dynamic changes of IgM and IgG were observed. There were no significant differences in IgG between groups of HIV viral load (*P* = 0·8650) (Fig. 3G), interestingly, IgM was significantly higher in individuals with low HIV viral load than in those with high HIV viral load. (*P* < 0·001) (Fig. 3H).

### The Correlation of Lymphocyte Subsets, SARS-CoV-2 Viral Load, and Antibody

**Fig. 4:**
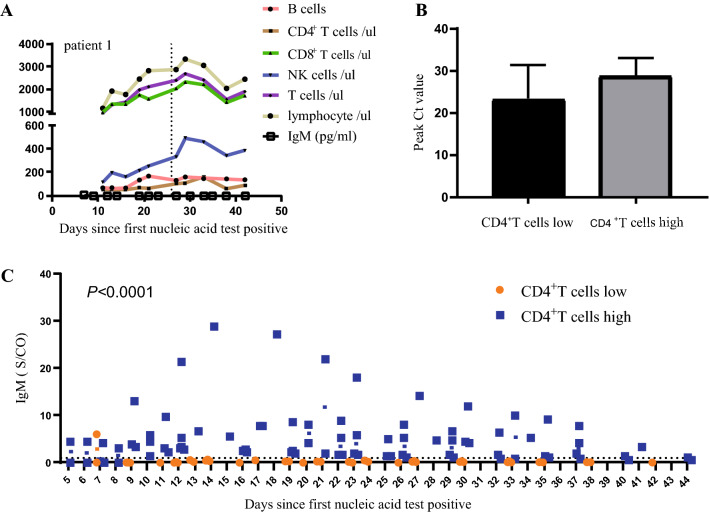
**A** shows the dynamic change characteristics of lymphocyte subsets of patient 1 over time, and the dashed line represents the time giving IL-2 treatment. Using 200 cells/µL as the limit, CD4^+^T cells are divided into high and low groups; comparing the differences in Peak Ct value of SARS-CoV-2 N gene (**B**) and IgM (**C**) between groups (**C**), respectively

Patient 1 had lower CD4^+^T cells with an average absolute count of 65(range 54·75–103) cells/µL, the median of B cells was 135(range 67·75–148·5) cells/µL, which was within the normal range; Interestingly, after IL-2 treatment, an upward trend of NK cells was observed over time; Lymphocytes, T cells and CD8^+^T cells also increased in different degrees; IgM antibody titers are consistently low. The CD4^+^T cells of 3 cases were less than 200 cells /100µL (Table 2); Taking 200 cells/µL as the limit, CD4^+^T cells were divided into “high” and “low” groups, the Peak Ct value of SARS-CoV-2 N gene was found there is no significant difference between groups of CD4^+^T cells. The single most striking observation to emerge from the data comparison was The IgM of “high” group was significantly higher than that of “low” group (Fig. 4).

**Table 2:** The average value of CD4+ T cells from 12 cases

	25% Percentile	Median	75% Percentile
Patient1	54·75	65	103
Patient2	24·25	27·5	29·25
Patient3	206	238	246
Patient4	227	255·5	284
Patient5	499·8	540	573·5
Patient6	287·5	345	424
Patient7	446·8	491·5	541·3
Patient8	30·75	48	58·25
Patient9	130·5	187·5	224·3
Patient10	211	272	294
Patient11	15·75	20	22·25
Patient12	490	597	857

### Dynamic Changes of the Measured Cytokines

**Fig. 5:**
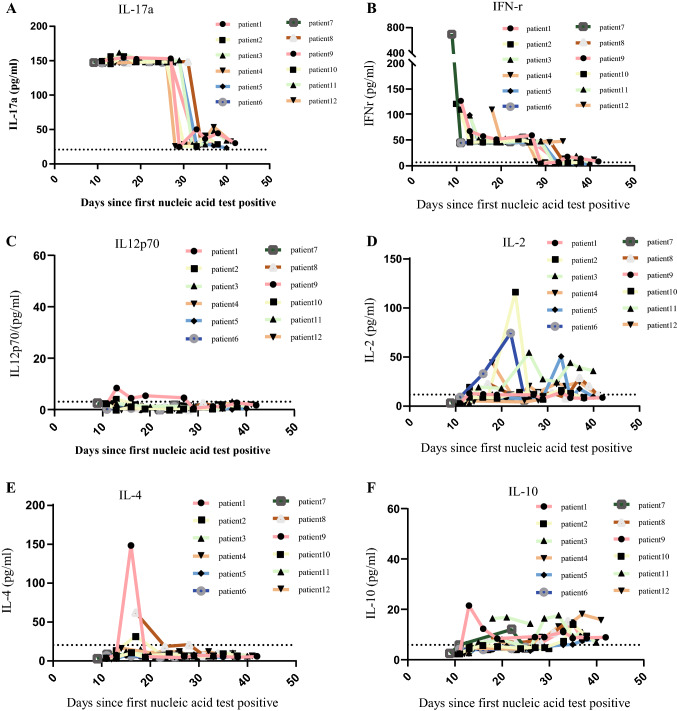
**A**–**F** represent the dynamic change of IL-17a, IFN-r, IL-12p70, IL-2, IL-4, IL-10, respectively. Dashed line represents the upper limit of the normal range

Since the first nucleic acid test is positive, the eight cytokines will be tested at the interval of 3–7 days, including IL-6, IL-12p70, IL-4, IL-17a, IL-10, IL-2, IFN-r, and TNFα. Interestingly, as the treatment progresses and the conditions of patients’ improvement, IL-17a and IFN-r had a downward trend. Until hospital discharge, IL-12p70, IL-2, IL-4, IL-10 always remained a low level (Fig. 5).

### Dynamic Change of Ferritin in All Cases

**Fig. 6:**
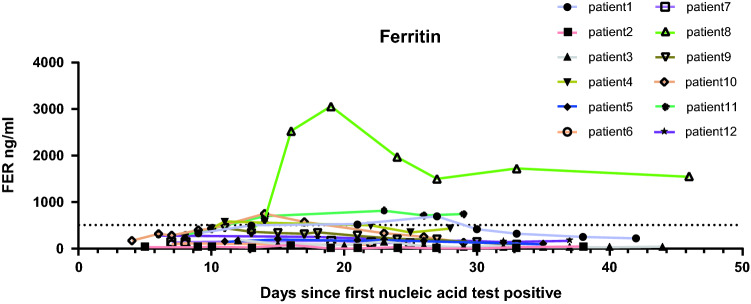
The dynamic change of ferritin (FER)over time. Dashed line in Y axis represents the upper limit of the normal range

Ferritin was high in 1 patient with NTM infection, while ferritin was maintained within the normal range in the remaining patients, which not much had changed (Fig. 6).

## Discussion

The epidemic was caused by the SARS-CoV-2 (Delta variant), which spread rapidly in Henan Province, China. Interestingly, Patients with COVID (Delta variant)/HIV co-infection had no typical characteristics of COVID-19, and their clinical symptoms do not change much from before the infection. Of those, only 2 cases required oxygen therapy through nasal cannula, the absorption of lesions in the chest CT imaging is fast and complete with no sequelae. To our knowledge, few studies have fully reported CT images of COVID-19/HIV co-infected patients before and after treatment, and researchers have been arguing about the clinical characteristics and prognosis of such patients [10–12, 14, 16, 34]. We collected all COVID-19 (Delta variant)/HIV co-infected patients in this epidemic for analysis and research, providing evidence to guide clinical practice and future research.

Previous studies only analyzed one of the 1ab gene and N gene of SARS-CoV-2, and did not combine them with the HIV viral load. However, our study dynamically observed and analyzed the changes of 1ab and N genes of SARS-COV-2 in these patients, as well as their relationship with HIV viral load. The data in this study found that Ct values were consistently low (Ct < 30) for about 21 days, indicating that patients with COVID-19(Delta variant)/HIV-coinfection had a high viral load and that the condition lasted longer. In this study, the virus shedding time (turn negative time) was calculated for these patients, with an average of 32·36 ± 2·643 days, longer than the turn negative time (21 days) for COVID-19 patients without infection with HIV [35, 36]. The correlation analysis between SARS-CoV-2 (Delta) and HIV viral load found that the viral load of SARS-CoV-2 has nothing to do with the viral load of HIV. However, the small sample size of our study (*n* = 12) is a limitation, the inferences need to be further tested with a larger sample size.

It was found in the dynamic detection of nucleic acid that Ct value would be 40 (nucleic acid negative) during the period of low continuity. It may be related to the sensitivity of the2019-nCoV Nucleic Acid kit and sampling error. In order to improve the positive rate and specificity of nucleic acid detection, we suggest that the sampling location should be standardized, that is, deep pharyngeal sampling, nasopharyngeal swabs and oropharyngeal swabs should be mixed, and two samples should be taken and sent to two different testing institutions for examination.

BRII-196 and BRII-198 are human IgG1 subtype antibodies, which are cocktail of COVID-19 antibodies for clinical emergency use [37]. We discovered that the sequential treatment with BRII-196 and BRII-198 significantly increased IgG levels in these patients. We also found that azivudine and convalescent plasma did not significantly increase IgG levels. Interestingly, azivudine and convalescent plasma significantly increased IgM levels in patients, while BRII-196 and BRII-198 were not effective for IgM. This finding is an unexpected and previously unreported observation, with potential therapeutic impact. Therefore, we recommend that patients with low IgM antibody levels should be given azivudine and convalescent plasma. Similarly, for patients with low IgG levels, BRII-196 and BRII-198 antibody cocktail combination regimens should be given to accelerate the elimination of Delta variant. However, this concept, which is based on this research, needs to be validated by large-scale future clinical trials.

Surprisingly, after patient 1 was given IL-2 treatment, the curve of NK cells over time showed an upward trend, the curve of lymphocytes, T cells, and CD8^+^T cells over time also increased slightly. The HIV viral load was divided into two groups (< 200 and ≥ 200 copies/ml), it was found that no significant difference between peak Ct value of SARS-CoV-2 N gene and CD4^+^T cell was evident, the data may indicate that there is no close one-to-one relationship between SARS-CoV-2 and CD4^+^T cells; However, the IgM of patients with high CD4^+^T cells was significantly higher than that of patients with low CD4^+^T cells. Based on this we recommended patients with humoral immune response disorders where CD4^+^T cells have always been at a low level and IgM antibody titers have always been low could be given IL-2 treatment, because IL-2 can enhance cell killing effect mediated by NK cells [38], It can enhance the innate immunity level and contribute to the elimination of SARS-CoV-2 [39]. This requires large-scale research to verify this conclusion.

After patients received anti-coronavirus treatment, IL-17a showed a significant downward trend, IFN-r also has a downward trend, IL-12p70, IL-2, IL-4 and ferritin remained at low levels. IL-17a and IFN-r are strong pro-inflammatory factors [40]. The immunodeficiency of patients living with HIV leads to the limited production of inflammatory factors, and after treatment, IL-17a, the only inflammatory factor that increased, also dropped significantly, this might lead to further containment of tissue damage.

We have found that the levels of IgM and IgG are low, combined with the characteristics of CT imaging, viral loads, and the inflammatory factors in these patients, we can infer that these features are associated with the pathogenesis of HIV and SARS-COV-2. HIV is a retrovirus [41], through the direct action of the virus and the cascade reaction of proteolytic enzyme, CD4^+^T cells appear pyroptosis, followed by immunodeficiency [42], Transition to systemic inflammatory disease mediated directly by the innate immune system, independent of the involvement of autoantibodies and T cells. Therefore, after HIV patients are infected with SARS-Cov-2, the production of IgM and IgG is obstructed, and the level of inflammatory factors is lower. SARS-CoV-2 is a positive-strand RNA virus, when a general person was infected by SARS-CoV-2, After completing cell invasion, genome replication, protein synthesis, virus assembly and release [43, 44], viruses are controlled by an early innate immune response, followed by a targeted adaptive immune response [45]. In the later stage, a large number of Th1-like cytokines were produced, causing effect of cell storm [46], finally resulting in inflammation, tissue damage, and accelerating the formation of ARDS in COVID-19 patients [47]. However, the immune deficiency of HIV patients may prevent COVID-19 patients from producing inflammatory storms, resulting in weaker lung damage, mild or even asymptomatic symptoms, faster and complete imaging recovery and other characteristics mentioned above. It had been observed that there was little relationship between patients' recovery and CD4^+^T cells, and IL-2 therapy is effective, possibly because IL-2 can stimulate the activity of NK cells. Therefore, it enhances innate immune function of the patient, increases the level of inflammatory factors in the body, and helps to clear virus. We suggested to continuously observe the changes in the levels of inflammatory factors and realize Individualized precision treatment according to the expression of inflammatory factors.

A limitation of this study is that the cases are too few (*n* = 12). Patients with this epidemic have more complications. Due to the characteristics of this epidemic, after matching age, gender, etc., no control group was found. Sample size in the present study was small, so large-scale clinical trials need to be performed to verify the generalizability of the present conclusion.

## Conclusions

Based on this study, we found that HIV infection might not exacerbate COVID-19 severity. Further large-scale clinical researches are needed to perform to justify that IgM levels were statistically higher in patients taking azvudine or convalescent plasma than in patients not taking these drugs. In addition, as treatment progresses, the levels of Cytokines and chemokines should be focused on.

## Supplementary Information

Below is the link to the electronic supplementary material.

Supplementary file1 (EMF 8 kb)

## Data Availability

Not applicable.

## Abbreviations

SARS-CoV-2: Severe acute respiratory syndrome coronavirus 2
COVID-19: Coronavirus disease 2019
WHO: World Health Organization
MERS-CoV: Middle East respiratory syndrome coronavirus
ACE2: Angiotensin-converting enzyme 2
AIDS: Acquired Immune Deficiency Syndrome
NTM: Nontuberculous mycobacteria
IFN: Interferon
IL: Interleukin
RT-PCR: Reverse transcriptase-polymerase chain reaction assays
Ct: Cycle threshold
HIV: Human immunodeficiency virus
CT: Computed tomography
PLWH: People living with HIV
S: The spike protein
TMPRSS2: The transmembrane protease serine 2
M-CLIAs: Magnetic particle chemiluminescence immunoassays
TNF: Tumor necrosis factor
